# Association between ionized calcium levels and 3-months mortality in geriatric patients hospitalized for COVID-19

**DOI:** 10.1016/j.jnha.2025.100661

**Published:** 2025-08-28

**Authors:** Audrey Boudaille, Alexis Bourgeais, Mathieu Corvaisier, Olivier Brière, Jennifer Gautier, Cédric Annweiler, on behalf of the GERIA-COVID study group

**Affiliations:** a UNIV ANGERS, Health Faculty, University of Angers, Angers, France; b Department of Geriatric Medicine and Memory Clinic, Research Center on Autonomy and Longevity, University Hospital, Angers, France; c UNIV ANGERS, UPRES EA 4638, University of Angers, France; d Department of Pharmacy, Angers University Hospital, 49933 Angers, France; e Gérontopôle Autonomie Longévité des Pays de la Loire, France; f Robarts Research Institute, Department of Medical Biophysics, Schulich School of Medicine and Dentistry, the University of Western Ontario, London, ON, Canada

**Keywords:** Calcium, COVID-19, Ionized calcium, Mortality, Older patients

## Abstract

**Objectives:**

The aim of this study was to determine an association between ionized calcium (CaI) levels and mortality 3 months after Covid-19 infection in geriatric population.

**Design:**

Observational retrospective cohort study.

**Setting and participants:**

One hundred and seventy-nine patients hospitalized in the geriatric acute care unit of Angers University Hospital were included. This specific unit specifically dedicated to COVID-19 patients was opened for 2 periods : from March 1, 2020 to June 30, 2020 and from October 1, 2020 to March 29, 2021. Covariates considered were: age, sex, Iso-Resources Groups (GIR) score ≤ 3, Parathyroid hormone (PTH), C-Reactive Protein (CRP), severe undernutrition defined as albumin < 30 g/L, history of cancer, history of cardiomyopathy, severe chronic renal failure (defined as clearance < 30 ml/min), Ordinal Scale for Clinical Improvement (OSCI) score, use of systemic corticosteroids and usual vitamin D supplementation. Direct potentiometry was also used as a covariate.

**Measurements:**

The time elapsed between the diagnosis of COVID-19 and death was studied by survival curves calculated using Cox model in each group defined by serum CaI concentration (>1.22 and ≤1.22 mmol/L).

**Results:**

In the Cox model, a significant association was found between CaI over 1.22 mmol/l and 3-months mortality, analyzed in an adjusted model (HR 2.40 [1.34–4.31], *p* = 0.003).

**Conclusion:**

There is an association between CaI and 3 months mortality in geriatric patients hospitalized for COVID-19.

## 1 Introduction

Coronavirus disease 2019 (COVID-19) has caused hundreds of thousands of deaths since it was first identified in Wuhan, China, in December 2019 [1]. This viral infection mainly affects the respiratory system. According to various studies, older age and common comorbidities worsen the prognosis of COVID-19 patients [2,3]. Since 2019, dyscalcemia has been established as a potential prognosis factor [4]. Hypocalcemia was found to be negatively correlated with inflammatory factors, and associated with increased in-hospital mortality [5,6]. Unlike hypocalcemia, the role of hypercalcemia is unclear.

Dyscalcemia has been observed in bacterial, viral pneumonia, particularly in cases of SARS-CoV virus and Ebola [7,8]. The virus uses calcium for many aspects of the replication cycle. Active uptake of calcium ions by mitochondria increase ATP production to meet the increased energy demands of continuous viral replication [9]. Moreover, experimental studies show that the SARS-CoV E gene encodes a small transmembrane ion channel protein permeable to Ca²^+^, which is highly expressed during infection. This disruption of calcium homeostasis may trigger inflammatory pathways, increasing levels of IL-1β, TNF, and IL-6 [10]. Circulating calcium accounts for 0.07% of total calcium. There are two measurable forms: ionized calcium (CaI) (40-50%) and calcium bound to proteins (50-60%), especially to albumin [11]. However, it remains the gold standard, CaI is not routinely measured in older patients and 30% of older patients could be misclassified with regard to their calcium status if albumin-corrected calcium is used [[12], [13], [14]]. The reasons for this discrepancy in the older population remain largely unknown. Few investigations have specifically addressed the potential role of normal-to-high calcium levels in the context of COVID-19 [15,16]. In 2021, an Angers University Hospital study found that a normal-to-high calcium concentration, estimated early in COVID-19 from corrected calcium levels, was associated with higher 3-month mortality in frail older patients [15]. CaI measurement could be more relevant than albumin-corrected calcium to properly estimate calcium status in the older population [13,14]. This study aims to strengthen the evidence of increased mortality for high or normal-high calcium values, and to better understand the factors influencing long-term complications in older patients with covid-19.

Within this context, the aim of the present study was to assess the association between normal-to-high CaI levels and 3-months mortality among older population diagnosed with COVID-19.

## 2 Material and methods

### 2.1 Study design and participant selection

This study is a retrospective cohort study based on the GERIA-COVID database. This was carried out in the geriatric acute care unit specifically dedicated to COVID-19 patients in the University Hospital of Angers, France (ClinicalTrials.gov NCT04560608). This specific unit was opened for 2 periods of time: from March 1, 2020 to June 30, 2020 and from October 1, 2020 to March 29, 2021. To be included, the patient and/or relatives had to have no objection to the use of anonymized clinical and biological data for research purposes. A total of 560 patients were included in GERIA-COVID.

### 2.2 Inclusion and exclusion criteria

**Fig. 1:**
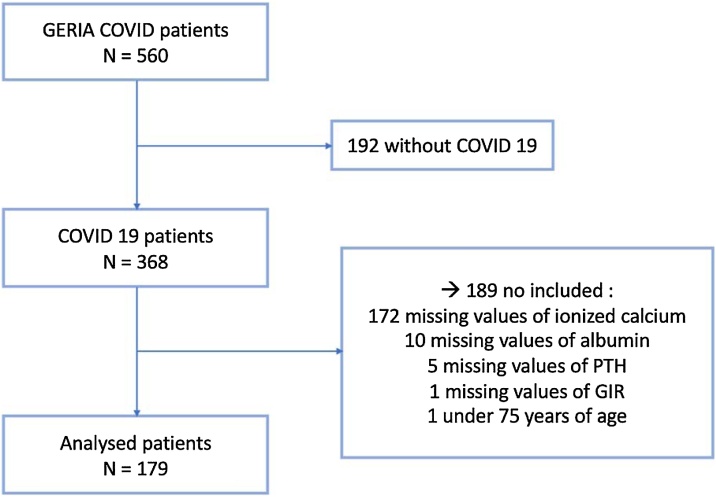
Flow chart.

The inclusion criterion for the present analysis was to be diagnosed COVID-19 using Reverse Transcriptase (RT) - Polymerase Chain Reaction (PCR) and/or chest Computerized Tomography (CT) scan (Fig. 1). The exclusion criteria were as follows:

1) Data unavailable on blood test (ionized calcium, albumin and Parathyroid hormone (PTH)) at hospital admission;
2) Data unavailable on Iso-Resources Groups (GIR);
3) Age under 75 years

### 2.3 Biological data and COVID-19 diagnostic

Blood samples were collected on admission to the geriatric acute care unit. All biological samples were analyzed at the biology department of the University Hospital Angers. COVID-19 was diagnosed with RT-PCR and/or chest CT-scan. CaI was collected with a blood gas syringe and analyzed using blood gas machine from the first of March 2020 to the 29th of November 2020 then by direct potentiometry (ABL 90 Flex plus or ABL 800) from the 30th of November 2020 to the 29th of March 2021. This measurement was systematically adjusted to pH. PTH and 25(OH)D were measured with an immunoassay method (Automate LiaisonXL, DiaSorin). The first dosage of CaI was performed on the 2nd of October 2020. C-Reactive Protein (CRP) and albumin were measured with immunoturbidimetric method (Advia Chemistry XPT, Siemens). Calcemia were measured with colorimetric method. Glomerular filtration rate has been estimated with Modification of Diet in Renal Disease.

### 2.4 Covariates

Age, sex, Iso-Resources Groups (GIR) score ≤ 3 (measured from 1 (highly dependent) to 6 (independent)), PTH, CRP, severe undernutrition (defined as an albuminemia inferior to 30 g/L), history of cancer, history of cardiomyopathy, severe chronic renal failure (defined as clearance < 30 ml/min), COVID severity (defined as an Ordinal Scale for Clinical Improvement (OSCI) score ≥ 6/8) use of systemic corticosteroids and usual vitamin D supplementation. Direct potentiometry was also used as a covariate. An OSCI score of 6 corresponds to an intubated patient.

### 2.5 Overall mortality

Main outcome was 3-months all-cause mortality. Follow-up began on the date of COVID-19 diagnosis for each patient. Vital status after 3, 6 months and 2 years was recovered by monitoring the National Institute of Statistics and Economic Studies (INSEE) register (https://www.insee.fr/fr/information/4190491).

### 2.6 Statistical analysis

A descriptive analysis of the participant’s characteristics was firstly performed using numbers (percentages) for qualitative variables and means ± standard deviations or medians [inter-quartile range (IQR)] for quantitative variables, as appropriate. Firstly, we presented participants according to the 2 groups defined by the serum concentration of CaI (>1.22 mmol/L and ≤1.22 mmol/L, this threshold corresponding to the highest tertile). Secondly, univariate Cox regression models were used to examine the associations of 3-month and 6-month mortality (dependant variable) with baseline clinical and biological characteristics (independent variables), including CaI. The proportional hazards assumption was assessed using Schoenfeld residuals and no significant violation was detected. All of the variables with a univariate p-value less than 0.20 as well as the clinical relevant variables according to the literature (History of cancer, Sever undernutrition, use of vitamin D supplementation or systemic corticosteroids) were included in a multivariate Cox regression model, then in a model with backward selection method with a significance level of 0.05 for variable retention. We chose to force the CaI measurement technique (direct potentiometry vs gas analyzer) into the model due to its potential role as a confounder. P-values <0.05 were considered significant. All statistics were performed using SAS® version 9.4 (SAS Institute Inc.).

### 2.7 Ethics statement

The study was conducted in accordance with the ethical standards set forth in the Helsinki Declaration (1983). No participant or relatives objected to the use of anonymized clinical and biological data for research purposes. Ethics approval was obtained from the Ethics Board of the University Hospital of Angers, France (2020/100). The study protocol was also declared to the National Commission for Information Technology and civil Liberties (CNIL; ar20-0087v0).

## 3 Results

### 3.1 Clinical characteristics of the population

Of the 179 participants, 124 have a CaI ≤ 1.22 mmol/l and 55 have a CaI > 1.22 mmol/l.

Ninety women (50.3%) were included; mean age was 86.4 ± 5.7 years. Sixty-two patients suffered from severe denutrition among the participants (34.6 %), 34 (19.0%) have history of cancer, 56 (31.3%) history of cardiomyopathy and 29 (16.2%) suffered from severe chronic renal failure. The median of PTH were 29.7 [18.7–43.6] pg/ml, the median of CRP was 63.0 [29.0–119.0] mg/l. Fifty three participants (29.6%) have OSCI score ≥ 6. Seventy four patient (41.3 %) used vitamin D supplementation and 124 patients (69.3 %) were treated with systemic corticosteroids (Table 1).

**Table 1:** Characteristics and comparisons of COVID-19 patients separated into two groups according to the study group (*N* = 179).

	Total cohort (*N* = 179)	Study group	
		Ionized calcium	Ionized calcium
		≤1.22 mmol/L (*n* = 124)	>1.22 mmol/L (*n* = 55)
**Demographical data**			
Age (years), mean ± E.T	86.4 ± 5.7	86.2 ± 5.4	87.0 ± 6.3
Female sex, *n(%)*	90(50.3)	62(50.0)	28(50.9)
GIR score ≤ 3, *n(%)*	105(58.7)	77(62.1)	28(50.9)
**Comorbidities**			
Severe undernutrition, *n(%)*	62(34.6)	46(37.1)	16(29.1)
History of cancer, *n(%)*	34(19.0)	28(22.6)	6(10.9)
History of cardiomyopathy, *n(%)*	56(31.3)	36(29.0)	10(36.4)
Severe chronic renal failure, *n(%)*	29(16.2)	24(19.4)	5(9.1)
**Hospitalization**			
OSCI ≥ 6, *n(%)*	53(29.6)	38(30.7)	15(27.3)
Serum PTH concentration (pg/ml), med[IQ]	29.7[18.7–43.6]	29.9 [18.2–44.1]	27.2 [20.0–38.9]
Serum CRP concentration (mg/L), med[IQ]	63.0 [29.0–119.0]	73.0 [32.5–146.5]	45.0 [22.0–81.0]
Duration of hospitalization (days), med[IQ]	10 [7–17]	10 [7–16]	11 [7–21]
**Treatment**			
Use of systemic corticosteroids, n(%)	124 (69.3)	94 (75.8)	30 (54.6)
Usual vitamin D supplementation, *n(%)*	74 (41.3)	50 (40.3)	24 (43.6)
Usual calcium supplementation, *n(%)*	12 (6.7)	9 (7.3)	3 (5.5)
Direct potentiometry, *n(%)*	101 (56.4)	84 (67.7)	17 (30.9)
**Follow-up**			
3-month mortality, *n(%)*	71 (39.7)	46 (37.1)	25 (45.5)
6-month mortality*, n(%)*	80 (44.7)	52 (41.9)	28 (50.9)
2-year mortality, *n(%)*	101(56.4)	64(51.6)	37(67.3)

Data presented as n (%) where applicable; COVID-19: Coronavirus Disease 2019; CRP: C-Reactive Protein; GIR: Iso Resource Groups; OSCI: Ordinal Scale for Clinical Improvement; PTH: Parathyroid hormone.

### 3.2 Association between CaI and 3-months mortality (Table 2)

In Cox proportional-hazards models, a significant association was found between serum CaI concentration > 1.22 mmol/l and 3-months mortality analyzed in fully-adjusted model (HR = 2.40 [1.34–3.41], *p* = 0.003). GIR score ≤ 3, OSCI ≥ 6, and Serum CRP concentration were associated with 3-months mortality in unadjusted model (*p < 0.001*) and remained significant in the fully-adjusted model (respectively, *p = 0.023, p < 0.001 and p = 0.012*). Age, history of cardiomyopathy, severe chronic renal failure, serum PTH concentration, serum CRP concentration and use of systemic corticosteroids were associated with 2 year mortality in unadjusted model but not in the fully-adjusted model.

**Table 2:** Multiple Cox proportional-hazards model showing the hazard ratio for 3-month mortality (dependent variable) among COVID-19 patients according to serum ionized calcium levels (independent variable), adjusted for potential confounders (*N* = 179).

	Unadjusted model		Fully-adjusted model		Model with backward selection method	
	HR [95% CI]	*p*	HR [95% CI]	*p*	HR [95% CI]	*P*
Ionized calcium > 1.22 mmol/L	1.28[0.79–2.09]	0.314	2.40[1.34–4.31]	**0.003**	2.29[1.35–3.89]	**0.002**
Direct potentiometry	0.92 [0.58–1.47]	0.731	1.18[0.66–2.10]	0.584	1.32[0.80–2.17]	0.286
Age	1.06[1.02–1.11]	**0.004**	1.03[0.97–1.09]	0.314		
Female sex	0.65[0.41–1.04]	0.075	0.87[0.50–1.52]	0.634		
GIR score ≤ 3	3.49 [1.97–6.19]	**<0.001**	2.12[1.11–4.04]	**0.023**	2.29[1.26–4.18]	**0.007**
Severe undernutrition	1.43[0.89–2.30]	0.136	0.84[0.50–1.41]	0.498		
History of cancer	1.07[0.59–1.91]	0.829	1.82[0.94–3.53]	0.077		
History of cardiomyopathy	1.75[1.09–2.82]	**0.021**	1.26[0.69–2.32]	0.451		
Severe chronic renal failure	3.02[1.81–5.05]	**<0.001**	0.95[0.47–1.97]	0.884		
OSCI ≥ 6	18.60[10.65–32.48]	**<0.001**	21.19[10.70–41.98]	**<0.001**	20.16[10.75–37.79]	**<0.001**
Serum PTH concentration (pg/ml)	1.008[1.003–1.013]	**0.004**	1.008[0.999–1.015]	0.052	1.009[1.003–1.014]	**0.004**
Serum CRP concentration (mg/L)	1.006[1.003–1.009]	**<0.001**	1.005[1.001–1.009]	**0.012**	1.006[1.002–1.009]	**0.001**
Use of systemic corticosteroids	2.11 [1.18–3.79]	**0.012**	1.44 [0.73–2.81]	0.290		
Usual vitamin D supplementation	0.72[0.44–1.18]	0.190	0.93[0.53–1.61]	0.793		

CI: confidence interval; CRP: C-Reactive Protein; GIR: Iso Resource Groups; OSCI: Ordinal Scale for Clinical Improvement; PTH: Parathyroid hormone; HR: hazard ratio.

Bold means p-values are significant (p < 0.005).

Serum CaI concentration > 1.22 mmol/l, GIR score ≤ 3, OSCI ≥ 6, serum PTH concentration and Serum CRP concentration were associated with 3-months mortality in model with backward selection (respectively, *p = 0.002, p = 0.007, p < 0.001, p = 0.004, p = 0.001*).

### 3.3 Association between CaI and 6-months mortality (Table 3)

In Cox proportional-hazards models, a significant association was found between serum CaI concentration > 1.22 mmol/l and 6-months mortality analyzed in fully-adjusted model (HR = 2.19 [1.26–3.83], *p = 0.006*). GIR score ≤ 3 and OSCI ≥ 6 were also associated with 6-months mortality in unadjusted model (*p < 0.001*) and remained significant in the fully-adjusted model (respectively, *p = 0.014* and *p < 0.001*). Age, history of cardiomyopathy, severe chronic renal failure, serum PTH concentration, serum CRP concentration and use of systemic corticosteroids were associated with 6-months mortality in unadjusted model but not in the fully-adjusted model.

**Table 3:** Multiple Cox proportional-hazards model showing the hazard ratio for 6-month mortality (dependent variable) among COVID-19 patients according to serum ionized calcium levels (independent variable), adjusted for potential confounders (*N* = 179).

	Unadjusted model		Fully-adjusted model			
	HR [95% CI]	*p*	HR [95% CI]	*p*	HR [95% CI]	*p*
Ionized calcium > 1.22 mmol/L	1.29[0.81–2.04]	0.280	2.19[1.26–3.83]	**0.006**	2.15[1.28–3.62]	**0.004**
Direct potentiometry	0.79[0.51–1.22]	0.280	0.98[0.57–1.68]	0.930	1.13[0.69–1.86]	0.622
Age	1.07[1.03–1.11]	**0.001**	1.03[0.98–1.09]	0.200		
Female sex	0.68[0.44–1.06]	0.089	0.86[0.52–1.44]	0.527		
GIR score ≤ 3	3.10[1.85–5.20]	**<0.001**	2.07[1.15–3.70]	**0.014**	2.54[1.48–4.35]	**<0.001**
Severe undernutrition	1.25[0.80–1.97]	0.327	0.76[0.47–1.25]	0.276		
History of cancer	1.18[0.69–2.01]	0.549	2.03[1.12–3.70]	**0.017**	2.15[1.21–3.82]	**0.009**
History of cardiomyopathy	2.02[1.30–3.14]	**0.002**	1.46[0.83–2.57]	0.185	2.27[1.42–3.63]	**<0.001**
Severe chronic renal failure	3.11[1.91–5.08]	**<0.001**	1.14[0.58–2.26]	0.599		
OSCI ≥ 6	14.97[9.03–24.81]	**<0.001**	18.37[9.73–34.67]	**<0.001**	18.53[10.38–33.07]	**<0.001**
Serum PTH concentration (pg/ml)	1.009[1.004–1.014]	**<0.001**	1.006[0.999–1.013]	0.133		
Serum CRP concentration (mg/L)	1.005[1.001–1.008]	**0.004**	1.004[1.000–1.007]	0.064		
Use of systemic corticosteroids	1.69 [1.01–2.82]	**0.047**	1.26 [0.70–2.27]	0.448		
Usual vitamin D supplementation	0.75[0.47–1.18]	0.210	0.88[0.53–1.46]	0.621		

CI: confidence interval; CRP: C-Reactive Protein; GIR: Iso Resource Groups; OSCI: Ordinal Scale for Clinical Improvement; PTH: Parathyroid hormone; HR: hazard ratio.

Bold means p-values are significant (p < 0.005).

Serum CaI concentration > 1.22 mmol/l, GIR score ≤ 3, OSCI ≥ 6, history of cancer and history of cardiomyopathy were associated with 6-months mortality in model with backward selection (respectively, *p = 0.004*, *p < 0.001, p < 0.001*, *p = 0.009* and *p < 0.001*).

## 4 Discussion

Two year mortality was significantly more frequent in the group with CaI over 1.22 mmol/l, in geriatric population with COVID-19, regardless of frailty, severe denutrition, initial severity, comorbidities and concomitant treatment influencing calcium metabolism. This finding suggests that geriatric patients hospitalized with COVID-19 and presenting with high-normal CaI levels have more than twice the risk of dying within 3 months compared to those with lower CaI levels. Such a magnitude of effect is clinically meaningful, especially in a population already vulnerable due to age and comorbidities. No studies have focused on the treatment of hypercalcemia in the context of COVID-19 to assess the benefit in terms of mortality.

To explain this result, the impact of cardiovascular risk after COVID-19, as observed with other viruses, is a plausible hypothesis [[17], [18], [19]]. In the older population, the elevated cardiovascular risk could contribute to the higher 3-month mortality, as this risk peaks within the first 3 months post-COVID-19 [18,20].

Calcemia can be exacerbated by immobilization, particularly in the older patients, and by isolation in a room for several days [21]. This was particularly the case in the first wave in France, as room visits, including physiotherapy, were limited. This may partly explain the high trend in calcium levels in the cohort patients.

SARS-CoV-2 infection also increase frailty and cardiovascular risk in older patients, notably by reducing physical activity and increasing inactivity [22]. Various factors, can also influence the frailty of older patients affected by COVID-19 such as lockdown, cardiovascular disease or neurocognitive impairment [23].

Independent association between CaI and mortality have been studied in hospitalized patients regardless of diagnosis. Contrast evidences exist on the association between hypercalcemia and mortality in healthy patients as some studies found a decrease in mortality [24]. Others observed a U-shaped relationship, indicating the importance of high and low trends in calcemia [6]. *Thongprayoon et al.* assessed in a large cohort study that CaI levels were correlated with hospital mortality and 1 year mortality (HR 1.47 [1.32-1.62], *p < 0.001*). In the referenced study, the upper limit of CaI associated with increased in-hospital and 1-year mortality was ≥ 5.20 mg/dL (1.297 mmol/L). Our study does not identify an identical threshold, due to a smaller sample size, which prevents participants from being stratified into several groups [6]. Moreover *Reid and al.* in a systematic review and metanalysis identifies an association between total calcium and long term mortality (mean follow up 13.4 years) with a HR of 1.13 [1.09–1.18] for 1 higher standard derivation of total calcium [19]. In the context of COVID-19, the magnitude of the association between calcium and long term mortality appears to be higher than in the general population.

In COVID patients, fewer studies have focused on the association of long term mortality and normal to high calcium levels. Corrected calcium report increased mortality at three months with adjusted calcium over 2.5 mmol/L [15]. Conversely, *Sun* et al. found no association between normal to high blood calcium levels and 28-day mortality. However, this association is observed at calcium concentrations below 2.0 mmol/L [25].

Certain limitations need to be emphasized. First, the majority of hospital admissions occurred during winter, a period when co-infections are possible but were not considered in our study. Second, vaccination status was not accounted for, although it could influence both initial and long-term outcomes. Few reports have theoretically explored the interaction between mRNA vaccination and calcium metabolism [26]. Third, our study focuses on patients from the first wave of the pandemic, when COVID-19 was poorly understood and circulating variants differed from those seen today. Since then, therapeutic strategies have evolved, particularly with the introduction of antivirals. Few studies have examined these therapies and their side effects; for instance, Ritonavir has not been associated with calcium disturbances [27]. Fourth, different analyzers were used to measure CaI via direct potentiometry although we accounted for this potential bias in our adjusted model. In addition, the CaI of the majority of patients was analyzed by the second analyzer. Fifth, we did not take into account calcium supplementation in adjusted model as there were 12 included patients under this treatment. Calcium supplementation could have an effect on long-term mortality due to the possible effect on cardiovascular risk and the development of neurocognitive disorders [28,29]. Finally, the small size of the population prevents these results from being generalized on a larger scale and may explain why certain covariates in the model have large confidence intervals (OSCI for example).

The strengths of our study are a robust primary endpoint, which is death. Few studies have focused on the CaI measurement in COVID-19. Moreover, few studies have taken normal-to-high values into account, and not just strict hypercalcemia, which challenges the interpretation of normal calcemia in the context of COVID-19. Possible cofounders have been taken into account in the adjusted model (notably dependency, severity, use of vitamin D supplementation or use of oral glucocorticoid). More studies on new form of SARS-CoV-2 are necessary to draw definitive conclusions on the results of this study.

## 5 Conclusion

In conclusion, CaI levels > 1.22 mmol/L may be associated with increased 3-month mortality in geriatric patients hospitalized for COVID-19. It should be noted that few studies have considered normal to high calcium values, which calls into question the current interpretation of “normal” calcium levels in the context of COVID-19. These findings suggest that clinicians could monitor CaI levels, even in the high normal range, in the older population with COVID-19 as a potential long term prognostic marker.

Further research, should be conducted to determine whether early identification and management of elevated CaI levels can improve outcomes, thereby guiding personalized therapeutic strategies in this vulnerable population.

## Funding

This research did not receive any specific grant from funding agencies in the public, commercial, or not-for-profit sectors.

## Disclosure statement

None declared.

## Data available statement

Patient level data are freely available at DRCI@chu-angers.fr to qualifying researchers registered with an appropriate institution and who submit a proposal with a valuable research question. There is no personal identification risk within this anonymized raw data, which is available after notification and authorization of the competent authorities.

## CRediT authorship contribution statement

**Audrey Boudaille:** Formal analysis, Validation, Visualization, Writing - original draft, Writing - review & editing. **Alexis Bourgeais:** Conceptualization, Formal analysis, Investigation, Methodology, Project administration, Validation, Visualization, Writing - original draft, Writing - review & editing. **Mathieu Corvaisier:** Writing - review & editing. **Olivier Brière:** Investigation, Writing - review & editing. **Jennifer Gautier:** Formal analysis, Investigation, Writing - review & editing. **Cédric Annweiler:** Conceptualization, Formal analysis, Investigation, Methodology, Supervision, Validation, Writing - original draft, Writing - review & editing.

## Declaration of competing interest

The authors declare that they have no known competing financial interests or personal relationships that could have appeared to influence the work reported in this paper.
